# Evidence of possible lower limb amputation in a tomb in an ancient Egyptian necropolis: the case report of an on-site radiographic analysis

**DOI:** 10.1259/bjrcr.20220090

**Published:** 2022-11-01

**Authors:** Carmelo Messina, Said Mahmoud Abd El-Moneim, Massimiliana Pozzi, Alice Tomaino, Lucie Biehler-Gomez, Marco Cummaudo, Cristina Cattaneo, Patrizia Piacentini

**Affiliations:** 1 Department of Radiology, IRCCS Istituto Ortopedico Galeazzi, Milan, Italia; 2 Dipartimento di Scienze Biomediche per la Salute, Università degli Studi di Milano, Milan, Italy; 3 Aswan and Nubia Antiquities Zone. Ministry of Tourism and Antiquities, Aswan, Egypt; 4 SCA - Società Cooperativa Archeologica, Milan, Italy; 5 Dipartimento di Studi Letterari, Filologici e Linguistici, Università degli Studi di Milano, Milan, Italy; 6 Laboratorio di Antropologia e Odontologia Forense (LABANOF), Sezione di Medicina Legale, Dipartimento di Scienze Biomediche per la Salute, Università degli Studi di Milano, Milan, Italy

## Abstract

**Objective:**

Despite the existing literature, the use of surgery to treat medical diseases in Ancient Egypt remains controversial. Regarding amputations, such procedures were performed in Egypt for therapeutic reasons, although they were never described in medical papyri. Here, we present the radiographic study of a possible lower limb amputation found in a recently discovered tomb in Aswan, Egypt.

**Methods:**

The necropolis is located on the west bank of the Nile, around the Mausoleum of the Aga Khan III. Around 45 mummies were found in the tomb, along with more than 400 mixed bones of adults and subadults. Radiographic analyses were carried out directly on-site in the proximity of the tomb using a digital portable device.

Among the mixed bones, we found a mature right femur with evidence of mid-diaphyseal bone interruption and exuberant reparative callus at the broken stump with woven periosteal new bone, indicating a recent and active healing process at the time of death. The X-ray study confirmed the presence of a mid-diaphyseal transverse fracture, highlighting the relatively sharp margins which were suggestive of a transverse cut at this point. A slightly radiopaque bone callus was visible as osseous spurs with circumferential and proximal directions; the exuberant bone callus revealed an ante-mortem trauma, suggesting the hypothesis of certain types of amputation. The X-ray showed no clear signs of other bone diseases or advanced taphonomic processes. Among the commingled remains, we also found the mature distal epiphysis of a right femur with radiographic evidence of extensive bone remodeling at the proximal broken stump. However, we cannot ascertain that these two femoral pieces corresponded to the same individual.

**Conclusions:**

Further studies will better clarify the causes of the bony lesion, which may be related to possible amputation of fracture from high-force blunt trauma. At present, the most likely cause relies on interpersonal violence, accidental occupation trauma and/or therapeutic treatment. Our report highlights how conventional radiology can still provide important results in the field of paleopathology, thanks to the possibility of using portable radiological devices directly on archaeological sites, thus overcoming technical difficulties in transporting bone mummified remains.

## Background

The ancient Egyptians were renown for their medical expertise and their different medical specialties have been reported.^1^ The existence of orthopedic diseases and possible treatments has been described in Ancient-Egyptian texts, such as therapeutic procedures to treat fractures or dislocated shoulder.^2^ In spite of this existing literature, the use of surgery to treat medical diseases in Ancient Egypt remains controversial: there are documents indicating little or no evidence of surgical procedures,^3^ while others report the use of surgical practices such that of trephination in dynastic Egypt.^4^

Regarding amputations, literature suggests that such procedures were performed in Egypt for therapeutic reasons, although they were never described in medical papyri.^5,6^ In this short report, we present the radiographic study of a possible lower limb amputation found in a recently discovered tomb in Aswan, Egypt.

**Figure 1.**
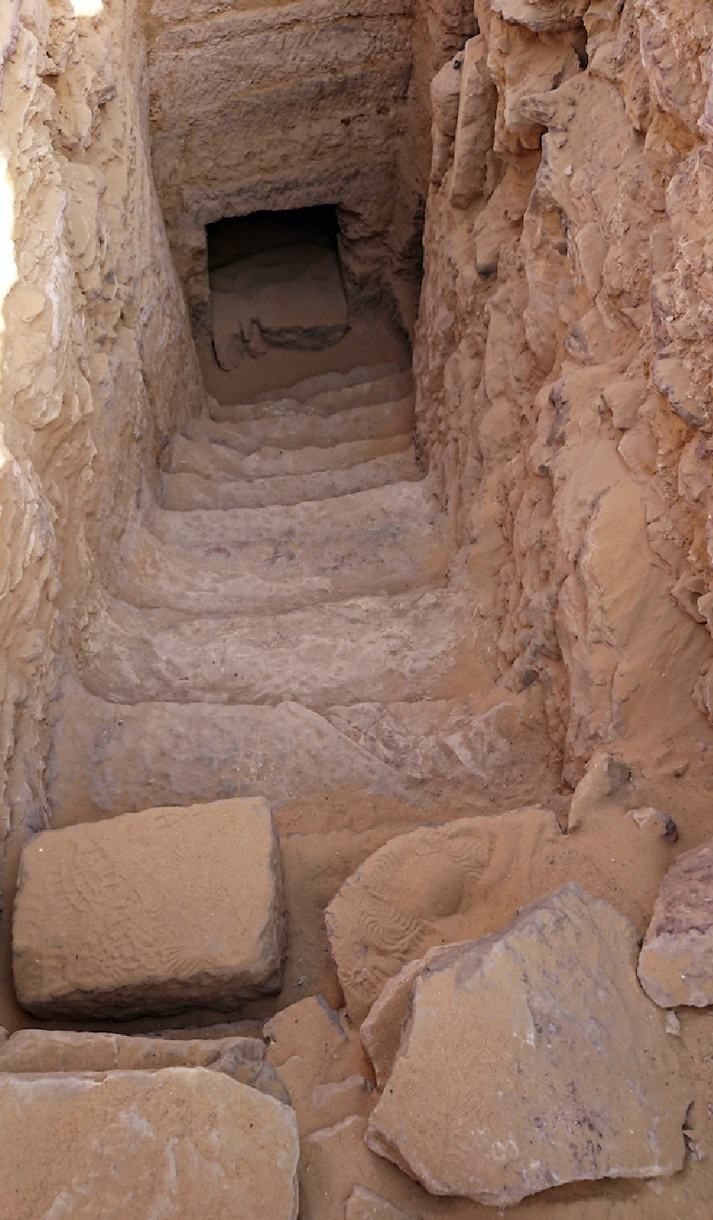
External view of the entrance of the tomb AGH026, during its excavation.

The necropolis where the tomb is located extends over 25.000 m^2^ on the west bank of the Nile, around the Mausoleum of the Aga Khan III. It is very likely that this is the necropolis where, for more than a millennium, the people who lived in Aswan from the Late Pharaonic to the Ptolemaic-Roman period (seventh century BC-3rd century AD) were buried.^7^ Around 300 tombs have been identified. Among them, in 2019, a hypogeic tomb designated “AGH026” was uncovered in a joint mission of the University of Milan and the Egyptian Ministry of Tourism and Antiquities in Aswan (Figure 1). Around 45 mummies in different states of preservation were found in the tomb, along with more than 400 mixed bones of adults and subadults (*i.e.,* immature individuals), some of them completely skeletonised.

Radiographic analyses were carried out directly on-site, in the proximity of the tomb, on the most relevant bone specimens and on some completely mummified individuals. X-rays were performed using a digital portable device (Rextar-X, Posdion), with a fixed tube voltage/current of 70kV/2mA, a focal spot of 0.4 mm and a target angle of 12°. We used a 14”x17” (350x 427.25 mm) Cesium flat panel detector, with a spatial resolution of 3.1 l p/mm.

## Case presentation

**Figure 2.**
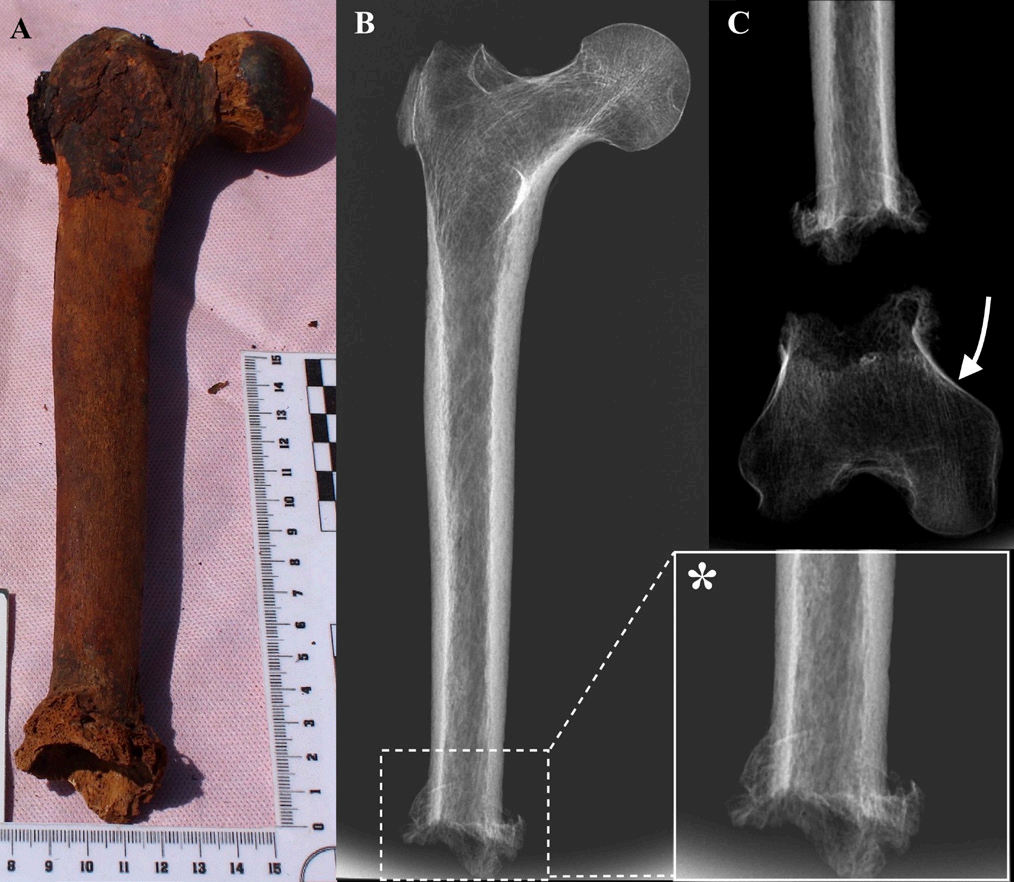
Frontal view of the right femur (on the left, (**A**)) and radiographic imaging (in the middle, (**B**)); note the mid-diaphyseal bone interruption, with slightly radiopaque bone callus visible as osseous spurs with circumferential and proximal directions (arrow). On the right (**C**), frontal radiograph of the right femur and distal femoral fragment found among the commingled remains (curved arrow). A transverse bone interruption is seen at the level of distal metaphysis with radiopaque bone callus, in a less extensive extent compared to the distal femoral diaphysis.

Among the mixed bones, we found a mature right femur (item n°28) 255.4 mm long with evidence of mid-diaphyseal bone interruption and exuberant reparative callus at the broken stump with woven periosteal new bone, indicating a recent and active healing process at the time of death (Figure 2). In addition, bone outgrowth in the form of osseous spurs with a proximal direction were visible on the anterior and posterior surfaces. Metric analyses of the maximum diameter of the femoral head indicated a female individual.^8^

The X-ray study confirmed the presence of a mid-diaphyseal transverse fracture which was straight across the bone, highlighting the relatively sharp margins which were suggestive of a transverse cut at this point. A slightly radiopaque bone callus was visible as osseous spurs with circumferential and proximal directions (Figure 2). The exuberant bone callus revealed an ante-mortem trauma, since the subject was alive long enough to ensure the formation of the bone callus itself, suggesting the hypothesis of certain types of amputation. At the point of fracture, the bone showed no signs of other diseases such as bone resorption or lytic areas suggestive of possible pathological fracture from an underlying infectious or tumoral disease. X-ray showed no clear evidence of hard callus due to long-lasting remodelling processes. The remaining femur did not show radiographic sign of metabolic disorders, nor advanced taphonomic processes. The head of the femur shows a regular articular surface, with only minimal sign of degenerative disease in the form of small superior pericephalic osteophytes.

Regarding the mature distal epiphysis we found among the commingled remains, the frontal X-ray showed a transverse bone interruption at the level of distal metaphysis with mild radiopaque bone callus, in a less extensive extent compared to what we found at the distal diaphysis. This was again suggestive of bone remodelling, without evidence of pre-existing pathological processes. Evidence of destructive taphonomic processes were found at the posterior aspect of medial condyle, leading to increased radiolucency at X-ray due to focal bone loss.

With regard to the differential diagnosis, several hypotheses may be raised to explain the bony lesion but two are the most probable. First, the lesion was the result of a transverse cut, probably from an amputation at the mid-diaphysis, to which the individual survived for some time resulting in a non-remodeled callus with active periosteal new bone. The presence of bone flaring in the callus with a proximal direction may be explained by the use of a tool acting as the remaining of the leg, like a prosthetic device, supporting the femur: the force applied by the tool on the amputated femur could have resulted in the observed new bone flaring pointed proximally. The second hypothesis is that a high-force blunt trauma caused a mid-diaphysis femoral fracture separating the bone in several pieces. The posterior breakaway spur on the proximal femoral fragment indicates in this hypothesis that the blunt force was applied anteriorly.

**Figure 3.**
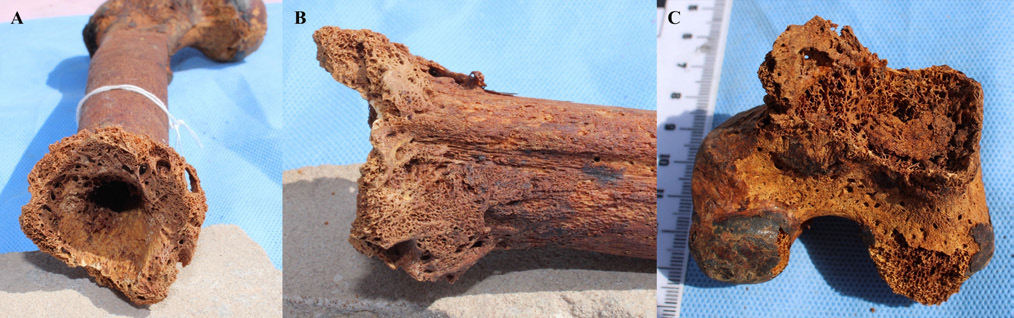
Photographic details of the lesion on the femoral shaft (left, A: distal view; middle, B: medial view, posterior is up) and distal femoral fragment (right, C: posteroproximal view)

Among the commingled remains, we found the mature distal epiphysis of a right femur (64.5 mm long) with extensive bone remodeling at the proximal broken stump. Metric analysis suggested that the fragment belonged to a female individual.^8^ However, given the commingling nature of the remains, we cannot ascertain that these two femoral pieces corresponded to the same individual. If they did, it is possible that failure of normal fracture healing resulted in a non-union of the bone fragments or pseudoarthrosis, which would explain the exuberant bone callus and the bone flaring. The third femoral fragment (the distal femoral shaft) which would have resulted from the blunt trauma was not recovered. This lack of recovery and the condition of the distal part makes the possibility of a fracture less probable given that there is no proof of the two parts belonging to the same individual. For both hypotheses, the female must have required help such as the use of clutches for walking. A photographic detail of the lesion on the femoral shaft and distal femoral fragment is presented in Figure 3.

Amputation may also be the consequence of a severely fractured bone for therapeutic purposes. In our case, a femoral fracture at this level would imply the hypothesis of a high-energy trauma (such as a fall from a great height, or severe blunt trauma), given the high mechanical resistance to fracture by the femoral diaphysis at this level, quite unlikely for a female in this case. Nevertheless, the Edwin Smith papyrus (one of the oldest known medical papyri) tells us that amputation as a treatment for fractures did not exist at that time, as they were usually treated with splinting methods.^9^ On the other side, several papers report cases of amputations as medical treatment both in the upper and lower limbs. The paper by Dupras et al^5^ presents four cases of amputation from the archaeological site of Dayr al-Barsha, Egypt: two cases of bilateral amputations of the feet (with healing pattern of the distal ends suggesting the use of prosthetic devices), one case of healed amputation of the left ulna close to the elbow, and a case of perimortem amputation of the distal end of the right humerus. According to the authors, in all four cases bone margins showed sign supporting the hypothesis that ancient Egyptians were using amputation as a form of medical treatment for possible diseases or traumatic injuries. Another case of possible forearm amputation at the level of ulna and radius is reported by D. R. Brothwell and V. Møller-Christensen in 1963.^10^ Although the authors did not exclude the possibility of an amputation due to social, legal of military determinants, they also stated that the finding may be a further indication of early Egyptian surgery (especially given that not just the hand was amputated, which reduces the weight of a punitive act).

Other tentative differential diagnosis hypotheses must consider the possibility of an amputation as a form of punishment. Such amputations were usually reported in the upper limb, but the punitive purpose remains controversial as it was considered a potential labour force deprivation for the society.^10^ Nevertheless, this hypothesis cannot be excluded as demonstrated by the discovery of a relief on the courtyard of the temple of Ramesses III at Medinet Habu, which shows the use of amputation as a punishment of prisoners: hands and penises of the enemies were brought to the king as symbol of victory.^11^

Finally, due to the crucial military role of this region in ancient times,^12^ another differential cause could be sought in a violent act of war. Considering that anthropological analysis revealed that this femur pertained to a female, we are inclined to rule out the possibility of a violent trauma occurred during a military fight. Still, the possibility that this female was injured during a conflict cannot be excluded.

## Conclusions

The first radiography of Ancient Egypt mummified remains was obtained only 3 months after the discovery of X-rays on November 8, 1895 by Wilhelm Conrad Roentgen.^13^ Since then, continuous radiology advancements led to an increasingly detailed and non-invasive study of mummies, especially since the introduction of computed tomography by the end of the 1970s.^14^

Our report highlights how conventional radiology is a tool that can still provide important results in the field of paleopathology, thanks to the possibility of using portable radiological devices directly on archaeological sites, thus overcoming technical difficulties in transporting bone mummified remains. We are carrying out additional ongoing studies, including the detailed analysis of the remaining commingled bones found on the site. Such studies will better clarify the causes of the bony lesion we found at distal femur, which is likely the result of an amputation or a displaced femoral fracture from high-force blunt trauma. At present, the most likely cause relies on interpersonal violence, accidental occupation trauma and/or therapeutic treatment.

## Learning points

- The presence of exuberant reparative callus on a broken mummy’s bone suggests an ante-mortem trauma
- X-ray study at the broken femur showed a mid-diaphyseal transverse fracture with sharp margins, suggestive of a transverse cut possibly from amputation
- The use of portable radiological devices directly on archaeological sites can still be of great value in the field of paleoradiology ·
